# Morbidity in early Parkinson's disease and prior to diagnosis

**DOI:** 10.1002/brb3.228

**Published:** 2014-04-08

**Authors:** Rune Frandsen, Jakob Kjellberg, Rikke Ibsen, Poul Jennum

**Affiliations:** 1 Department of Clinical Neurophysiology, Danish Center for Sleep Medicine, Glostrup HospitalGlostrup, Denmark; 2 Danish Institute for Health Services ResearchCopenhagen, Denmark; 3 itracksKlosterport 4E, Aarhus, Denmark; 4 Faculty of Health Sciences, Center for Healthy Aging, University of CopenhagenCopenhagen, Denmark

**Keywords:** Morbidities, National Patient Registry, Parkinson's disease, prediagnostic

## Abstract

**Background:**

Nonmotor symptoms are probably present prior to, early on, and following, a diagnosis of Parkinson's disease. Nonmotor symptoms may hold important information about the progression of Parkinson's disease.

**Objective:**

To evaluated the total early and prediagnostic morbidities in the 3 years before a hospital contact leading to a diagnosis of Parkinson's disease.

**Methods:**

Retrospective morbidity data from Danish National Patient Registry records (1997–2007) of 10,490 adult patients with a secondary care diagnosis of Parkinson's disease were compared with 42,505 control cases.

**Results:**

Parkinson's disease was associated with significantly higher morbidity rates associated with conditions in the following categories: mental and psychiatric, nervous system, gastrointestinal, musculoskeletal system and connective tissue, genitourinary, abnormal clinical and laboratory findings, injury, poisoning and certain other external causes, and other factors influencing health status and contact with health services. It was negatively associated with neoplasm, cardiovascular, and respiratory diseases.

**Conclusions:**

Patients with a diagnosis of Parkinson's disease present significant differences in morbidities early on, following, and prior to, their diagnosis, compared with healthy controls.

## Introduction

Parkinson's disease (PD) is a serious neurodegenerative disorder that affects a significant proportion of the adult population (Wickremaratchi et al. 2009; McCrone et al. 2011). PD leads to a deterioration in motor, mental, and functional skills and is associated with significantly raised mortality rates (Guttman et al. 2001). It is chronic and associated with serious negative impacts on patients' social life, family, quality of life, work, and health (Diem-Zangerl et al. 2009). Known comorbidities include sleep disturbances (Suzuki et al. 2011), depression (Dissanayaka et al. 2011), dementia (Aarsland and Kurz 2010a), falls and fractures (Duncan et al. 2012), and impulse control disorders (Djamshidian et al. 2011).

Significant progress has been made toward understanding the underlying pathophysiology (Weintraub et al. 2008; Bartels and Leenders 2009; Montgomery 2009), and improving the diagnostic accuracy (Montgomery 2006), and management (Olanow et al. 2009) of the disease. The underlying pathophysiology includes progressive destruction of multiple brain regions, especially, initially, the brain stem, the basic forebrain, the extrapyramidal system, and, in later stages, the cortical areas (Braak et al. 2003a). This progression is known as the Braak Staging Scheme for PD (Braak et al. 2003b; Dickson et al. 2010). Recent studies have highlighted the importance of symptoms and clinical findings before a diagnosis of PD (Postuma et al. 2012). However, the general population study of the total morbidity in early PD and before diagnosis of PD has not been systematically described. The disease is thought to have a long preclinical stage, so important information about the disease may go unnoticed in the period before diagnosis. A difficulty in studying early changes is the variable time between the onset of symptoms and contact with a doctor who can make the diagnosis of PD. The aim of this paper was to address the entire range of morbidities as early in the course of the disease as is possible with the current data. The data were based on PD diagnoses from the Danish National Patient Registry (NPR).

## Methods

### Subject selection

All patient hospital contacts in Denmark are recorded in the NPR by type and date of contact. The NPR includes administrative information, primary and secondary diagnoses, diagnostic procedures, and treatment procedures using the International Classification of Diseases (ICD-10) and their date. Specific clinical information, such as the UPDRS score and imaging results, is not present in the NPR. The NPR contains diagnoses from private and public hospitals, but does not record diagnoses from general practice.

Using the NPR, we identified all patients at least 20 years of age who were diagnosed with PD between 1997 and 2007. For the PD diagnoses, we used the code G20.9. The code G20 is not accepted in the NPR, so all PD patients are registered as G20.9 (Paralysis agitans). Hospital doctors report the NPR at the time of diagnosis. Then, using data from Denmark's Civil Registration System Statistics, we randomly selected citizens of the same age, gender, and marital status as the patients who did not have PD. Parity of socioeconomic status (SES) was ensured by selecting control subjects from the same part of the country as where the patient lived. The ratio of control subjects to patients was 4:1. Data from patients and matched control subjects who could not be identified in the Coherent Social Statistics database were excluded from the sample. More than 99% of the observations in the two groups were successfully matched. Morbidity data from the patients and matched control subjects were gathered from their year of diagnosis until 2007.

### Data analysis

Data were analyzed by developing a conditional logit model, where the dependent variable was the case–control group and the explanatory variables were dummies for the 21 major ICD10 diagnosis groups, omitting the group with no diagnosis 3 years before diagnosis. A second analysis included dummies for ICD10 diagnosis that occurred in more than 1% of either the case or control group. ICD10 diagnoses accounting for 1% or fewer of the total diagnoses were included in the main diagnosis groups. Only estimates for the ICD10 diagnoses are reported in the results, but the dummies for the main groups (including the remaining diagnoses) were included in the regression. Patients could be classified in more than one diagnostic group or with an ICD10 diagnosis during the 3 years before the diagnosis.

Not all patients had a 3-year observation period before the registered diagnosis; for the first 3 years of the period, the patient only had data for 1 or 2 years, but as this was also the case for the control group, we have included these shorter periods in the analysis.

Information before the PD diagnosis was extracted from the database for the years 1997–2007. Morbidity data were extracted as primary and secondary diagnoses and subdivided into main disease groups, in accordance with the World Health Organization (WHO) criteria for ICD-10. Odds ratios (ORs) and 95% confidence intervals (CIs) were calculated for the main diagnoses. For the main diagnostic groups that showed significant differences, we performed subgroup analyses to determine the most common diagnoses in each category that were associated with a PD diagnosis. We used a threshold of 1%, values greater than which were taken to indicate that diseases were selected. Odds ratios were then calculated for each of them.

Statistical analyses were done using SAS 9.1.3 (SAS, Inc., Cary, NC). The study was approved by the Danish Data Protection Agency. Data were handled to ensure personal anonymity, so neither individual nor ethical approval was required.

## Results

We identified 10,490 patients with PD and compared their data with those of 42,505 control subjects, aged more than 20 years. The age distribution of the patients and control subjects is shown in Table 1.

**Table 1:** Age and gender distributions of patients at the time of diagnosis and matched controls

	PD
Total/women (%)	10,490/4,721 (45)
Age, years	
<20	0 (0.0)
20–29	10 (0.1)
30–39	37 (0.3)
40–49	156 (1.5)
50–59	615 (5.9)
60–69	1,801 (17.2)
70–79	3,969 (37.8)
≥80	3,902 (37.2)

Data are presented as frequencies, with percentages in parentheses. The 10,490 patients with Parkinson's disease (PD) were matched with 42,505 control subjects.

### Morbidity before PD Diagnosis

The morbidities, subdivided into percentage incidence and OR with 95% CI, are shown in Table 2 for diagnoses made in more than 1% of PD patients (Table 3).

**Table 2:** Morbidities 3 years before diagnosis of Parkinson's disease, by major disease groups

	Parkinson's disease					
	
	Cases	Controls	Odds ratio statistics			
			
Disease group	%	%	OR	95% CI		*P*
Infectious and parasitic diseases	2.03	1.63	1.02	0.88	1.19	0.755
Neoplasms	6.41	6.65	0.85*	0.78	0.93	<0.001
Blood and immunological diseases	1.48	1.28	1.01	0.85	1.20	0.924
Endocrine, nutritional, and metabolic diseases	4.63	3.65	1.09	0.99	1.21	0.079
Mental and psychiatric disorders	3.00	1.29	1.74*	1.53	1.98	<0.001
Nervous system disorders	5.45	2.58	1.72*	1.56	1.89	<0.001
Diseases of the eye and adnexa	8.25	7.68	1.04	0.97	1.13	0.262
Ear, nose, and throat diseases	8.91	9.20	0.93*	0.86	1.00	0.041
Circulatory/cardiovascular diseases	15.00	15.60	0.82*	0.77	0.87	<0.001
Respiratory diseases	5.63	5.58	0.91*	0.83	0.99	0.032
Gastrointestinal diseases	11.39	8.94	1.16*	1.09	1.24	<0.001
Skin and subcutaneous tissue diseases	2.03	1.75	0.99	0.86	1.15	0.940
Musculoskeletal system and connective tissue diseases	12.27	9.98	1.12*	1.05	1.19	<0.001
Genitourinary diseases	10.07	6.82	1.31*	1.22	1.41	<0.001
Pregnancy, childbirth, and puerperium	0.05	0.08	0.49	0.18	1.33	0.164
Certain conditions originating in the perinatal period	0.01	0.00	0.93	0.10	9.15	0.953
Congenital malformations, deformations, and chromosomal abnormalities	0.21	0.15	1.12	0.71	1.78	0.629
Abnormal clinical and laboratory findings	14.16	10.08	1.26*	1.18	1.34	<0.001
Injury, poisoning, and certain other external causes	24.98	17.92	1.36*	1.29	1.43	<0.001
External causes of morbidity and mortality	0.02	0.00	3.10	0.41	23.45	0.274
Other factors influencing health status and contact with health services	27.03	20.94	1.22*	1.16	1.29	<0.001

* *P* < 0.001.

**Table 3:** Parkinson's disease: Morbidities 3 years before PD diagnosis. Only diseases occurring at a frequency of more than 1% are included

	Parkinson's disease						
	
		Cases	Controls	Odds ratio statistics			
				
Disease group	Diagnosis	%	%	OR	95% CI		*P*
Diseases of the eye and adnexa	Cataract H259	4.07	4.06	0.99	0.89	1.11	0.904
Diseases of the ear and mastoid process	Presbyacusis H911	5.04	5.33	0.94	0.85	1.03	0.173
	Hearing loss H919	1.33	1.36	0.92	0.77	1.11	0.389
Diseases of the circulatory system	Hypertension I109	1.30	1.27	0.92	0.77	1.11	0.390
	Angina I209 + I251	3.14	3.67	0.89	0.78	1.01	0.067
	Myocardial infarction I219	0.61	1.01	0.62^*^	0.48	0.81	<0.001
	Atrial fibrillation I489	1.99	2.24	0.84^*^	0.73	0.98	0.025
	Cardiac insufficiency I509	1.10	1.17	0.98	0.80	1.20	0.827
	Stroke I649 + I694	2.97	2.30	0.96	0.84	1.10	0.581
	Arteriosclerosis (extremities) I702	1.05	1.22	0.81^*^	0.66	0.99	0.038
Diseases of the respiratory system	Pneumonia J189	2.57	2.03	1.12	0.98	1.29	0.093
	Chronic obstructive pulmonary disease J449	0.92	1.33	0.65^*^	0.52	0.81	<0.001
Diseases of the digestive system	Inguinal hernia K409	1.85	1.50	1.16	0.99	1.36	0.059
	Constipation K590	1.23	0.59	1.57^*^	1.29	1.92	<0.001
Diseases of the musculoskeletal system and connective tissue	Lumbar pain/lumbar stenosis M480	1.03	0.45	1.59^*^	1.28	1.98	<0.001
Diseases of the genitourinary system	Urinary infection N300	1.22	0.66	1.30^*^	1.06	1.59	0.010
	Prostatic hypertrophy N409	3.69	2.53	1.30^*^	1.15	1.46	<0.001
Symptoms, signs and abnormal clinical and laboratory findings not classified elsewhere	Abdominal pain R108	1.04	0.72	1.15	0.93	1.42	0.208
	Hematuria R319	1.11	1.12	0.86	0.70	1.05	0.138
	Retentio urinae R339	1.53	1.02	1.13	0.94	1.35	0.201
	Lipothymia R559	2.36	1.44	1.31^*^	1.14	1.51	<0.001
Injury, poisoning and certain other consequences of external causes	Head trauma S010 + S019	2.47	1.06	1.78^*^	1.54	2.05	<0.001
	Fractures, extremities S422	1.29	0.78	1.34^*^	1.11	1.63	0.002
	Fractura radii, extremitas distalis S525	1.61	1.07	1.25^*^	1.06	1.48	0.010
	Contusion of hip S700	1.46	0.76	1.29^*^	1.07	1.55	0.007
	Contusion of shoulder S720	2.64	1.20	1.69^*^	1.47	1.94	<0.001
	Fractures, distal upper extremities S721	1.11	0.71	1.09	0.88	1.35	0.414
Factors influencing health status and contact with health services	Observation Z031 + Z039	10.06	7.50	1.16^*^	1.08	1.25	<0.001
	Observation of neurological disease Z033	3.95	1.01	2.57^*^	2.28	2.89	<0.001
	Observation of ischemic heart disease Z035	2.93	2.46	1.08	0.95	1.23	0.212
	Observation of urinary disease Z038	1.66	1.16	1.16	0.98	1.38	0.086
	Control after surgery Z090	1.97	2.17	0.80^*^	0.69	0.94	0.005
	Rehabilitation after trauma Z508 + Z509	2.29	1.42	1.09	0.94	1.27	0.266

Main diagnosis groups omitted from output. ^*^P < 0.001.

Parkinson's disease was positively associated with the presence of mental and behavioral disorders (OR = 1.74), diseases of the nervous system (OR = 1.72), digestive system (OR = 1.16), musculoskeletal system and connective tissue (OR = 1.12), and genitourinary system (OR = 1.31), symptoms, signs and abnormal clinical and laboratory findings not classified elsewhere (OR = 1.26), injuries, poisonings and certain other consequences of external causes (OR = 1.36), and other factors influencing health status and contact with health services (OR = 1.22).

PD was negatively associated with: neoplasm (OR = 0.85), diseases of the ear, nose, and throat (OR = 0.93), the circulatory system (OR = 0.82), and respiratory system (OR = 0.91).

## Discussion

The current data show that symptoms other than classic Parkinsonian symptoms are present early PD and perhaps before the latter type are dominant enough to enable diagnosis. This study used a national database to identify all diagnoses before hospital-registered diagnosis of PD in a controlled design. We show that nonmotor diagnoses include a wide range of disease areas, including genitourinary, digestive, neurological, and psychiatric disorders, and, notably, are associated with a significantly higher risk of falls/injuries. A lower incidence of morbidity was seen in PD in the neoplasms and cardiovascular disease groups. These data support the hypotheses that PD patients exert nonmotor symptoms and morbidities in the early years after a diagnosis and in the years before a diagnosis.

The genitourinary system diseases manifested themselves as prostatic hypertrophy (OR = 1.30) and increased urinary infection (OR = 1.30), which we believe is caused by autonomic dysfunctions (Winge and Fowler 2006).

The effects on the digestive system consisted of more frequent constipation (OR = 1.57), suggesting decreased gastrointestinal mobility probably due to dysfunctional autonomic activity (Winkler et al. 2011), associated with the accumulation of alpha-synuclein in the intestinal neurons (Lebouvier et al. 2009).The associations of PD with mental and behavioral disorders are well-known and represent other, nonmotor control areas of the brain affected by neurodegeneration. We found that PD was associated with mental and behavioral disorders prior to diagnosis. Depression and cognitive complaints have been reported (Dissanayaka et al. 2011), and are probably due to the early involvement of the raphe nuclei. No single diagnosis in the mental and behavioral disorders group had a frequency of above 1% in either group and was, therefore, excluded in the dataset.

A particularly interesting finding was the significantly higher risk of falls before diagnosis, even when we adjusted for age, gender, and social factors. Accidental falls are common in the elderly (Gillespie et al. 2009). Due to the type of motor, nocturnal, and autonomic involvement, we would expect truncal instability for PD to result in more falls and accidents. Even before diagnosis, PD patients were more likely to experience head traumas (OR = 1.78). We have no information about the cause of accidents (e.g., while supine, nocturnal, etc.), but we suggest that the high incidence of injuries (Duncan et al. 2012) may be attributed to the combined effect of autonomic dysfunction, nocturnal motor/behavioral (REM sleep behavioral disorder) (Suzuki et al. 2011), motor involvement with truncal instability, and cognitive involvement (Aarsland and Kurz 2010b). Another important consequence is that physicians should be aware of potential neurodegenerative disorders in patients who present with falls and injuries, especially if the injuries are serious, for example, to the head and face. It should be noted that we cannot rule out the possibility that a head trauma in itself increases the risk of developing PD, as has been proposed elsewhere (Goldman et al. 2012). However, if we include other injuries (hip, shoulder, face, etc.), as is possible in this study, the causal route is most likely that the increase in falls is caused by early truncal instability, autonomic dysfunction, and slow reaction time that predates the development of symptoms severe enough to be categorized as PD. This is supported by previous studies of the Danish PD population (Rugbjerg et al. 2008). Unfortunately, we have little information regarding sleep diagnosis in the current database. REM Sleep Behavior Disorder has a ICD-10 diagnosis code, but the low level of awareness about this disorder leads to poor registration and underestimation of the occurrence of sleep problems and diseases.

Another notable finding was the reduced incidence of cardiovascular diseases before the hospital diagnosis of PD compared with controls. This association was most pronounced for myocardial infarction (OR = 0.62). No effect of protection against myocardial infarction has been proposed or evaluated before. However, several factors could account for this finding, including the occurrence of lower blood pressure due to autonomic denervation in PD (Goldstein et al. 2000), changes in lifestyle factors etc. On the other hand, the prevalence of hypertension was the same in PD as in the controls. All protective lifestyle factors are of very small effect, resulting in only a small bias in any correlation in selection on the basis of PD (Wirdefeldt et al. 2011). The inability of population studies like this to correct for lifestyle bias is a clear weakness but it cannot be addressed with the data currently available. Another mechanism may be the involvement of the autonomic system, for example, autonomic dysfunction may protect against myocardial infarction. Reduction in or the removal of the cardiac artery's contractive reflex, seen in spasm angina during a myocardial infarction (Maseri et al. 1978; Conti 1984), should protect PD patients from serious cardiac events (Inazumi et al. 2000). However, no study has been done to establish whether such an effect exists. We cannot discount the possibility that PD cardiac events are underestimated in PD patients due to their generally high comorbidity and mortality rates. The finding requires replication and confirmation.

Lower incidences of neoplasm have been reported in PD patients (Bajaj et al. 2010). We found the correlation to be weaker than in the population case–control study of cancer prior to PD by D'Amelio et al. (D'Amelio et al. 2004), who found cancers in 6.8% and 12.6% of PD patients and matched controls, respectively. Our study examined a larger population and was based on factual hospitals reports, rather than being questionnaire-based, and so was not susceptible to any recall bias. We could not confirm the former finding because we found lower incidences of neoplasm before PD diagnosis. We did not differentiate between benign and malignant diseases.

Our study has several limitations: (1) Only diagnoses made in the hospital sector were included, for which reason we cannot conclude that the findings concerning changed morbidity prior to PD. The PD group is a mixture of prediagnostic PD and early PD patients from the 3 years before their hospital-registered diagnosis. (2) Clinical examination and diagnostic procedures have varying diagnostic accuracy. This has been addressed in the Danish Parkinson's disease population by Wermuth et al., who found that 82% of PD patients were correctly diagnosed in accordance with the strict United Kingdom Parkinson's Disease Society Brain Bank criteria (Wermuth et al. 2012). They also reported that 2.4% of patients in the Danish PD population had multiple system atrophy (MSA). The Wermuth study is a systematic review of all available data from 1040 patients seen in a hospital setting, collected by six neurological departments. When the diagnosis is made exclusively by movement disorder specialists diagnostic accuracy can be improved to 90% (Hughes et al. 2002), but this is difficult to achieve in population studies. (3) Confounder variables (e.g., BMI, smoking behavior, and other cardiovascular risk factors) were not recorded; symptoms and results of clinical evaluations (e.g., MRI, molecular imaging and UPDRS) were not considered because there is no national registry of such clinical data. Smoking has been found negatively associated with the presence of PD, although a causal relation has been doubted. It is a clear weakness that we cannot correlate for smoking as it is a factor that is negatively correlated with PD. A smaller proportion of smokers among PD patient could in part explain the smaller risk of myocardial infarction. However, no differences were indentified in hypertension, angina, cardiac insufficiency, or stroke. PD patients presented lower occurrence of arteriosclerosis of the extremities which support that that lifestyle might be the correlating factor.

As in other studies, there is a diagnostic difficulty differentiating MSA from PSP in the clinical setting (Wenning et al. 2011). The strength, however, is that our study included almost all (>99%) national PD cases with a hospital contact. The study considers the population statistics of morbidity in a comprehensive and nationally representative way that cannot be done in a single specialty center study. This makes the study applicable to the factual premorbid associations of patients diagnosed with PD in real-life clinical settings. All comorbidity data have the same deficiencies as PD data in that they come from the national database, and diagnoses recorded in the database come from individual doctors working throughout the entire Danish medical care system.

In Denmark, all patients with a hospital contact are registered in a National Patient Registry (NPR). Consequently, all those in Denmark with a diagnosis of PD and comorbidities are identified. PD is diagnosed in both the primary sector and in the hospital setting. The great strengths of the NPR are that it is a national database that includes all patients, it is time-locked (all reports must be associated with the patient contacts), and it encompasses a substantial follow-up time.

Healthcare in Denmark is free with respect to primary sector, specialist, and hospital care and diagnostics, thereby avoiding most of the possible sources of economic bias. This study aimed to address morbidity in early PD and prior to a diagnosis coinciding with the main WHO diagnostic groups.

As in other studies addressing PD, patients submitted to specialist and hospital sector are included. PD is generally a disease which due severity is diagnosed by specialist with contacts to the hospital sector one or more times. We cannot exclude that some patients with modest symptoms are unidentified, but generally the NPR are time-locked and complete in respect to identification of patients.

As PD is a disease without sudden onset, marking the start of the disease as the time of diagnosis is of course an approximation. In a previous study, we showed that PD patients had increased health care usage and social consequences up to at least 8 years before diagnosis. There is often a very long diagnostic delay between the onset of minor symptoms and the final diagnosis. We recognize that these data related only to prediagnoses but not to pre-Parkinsonian symptoms. The 3-year window proves that the other symptoms are at least not late symptoms of PD but rather identify them as arising at the beginning of the disease.

## Conclusion

Several results from this study confirm previous findings that patients with PD suffer from significant prediagnostic and early PD morbidities affecting genitourinary, digestive, neurological, and psychiatric conditions, and experience a significantly higher risk of falls/injuries. We found lower incidence of neoplasms and cardiovascular diseases. Consequently, patients with PD present a wide range of symptoms before diagnosis and early on in the disease. These findings may have implications for the future identification of earlier stages of PD disease.
